# Supplementary material for: HIVprotI: an integrated web based platform for prediction and design of HIV proteins inhibitors
Source: J Cheminform. 2018 Mar 9;10:12. doi: 10.1186/s13321-018-0266-y (PMC5845081; doi:10.1186/s13321-018-0266-y)
Supplement: Supplementary file 2 — Additional file 2. Source code of HIVProtI web server. [file 13321_2018_266_MOESM2_ESM.zip › HIVprotI_Source-code/hivprotI/weka-3-6-6/doc/weka/core/xml/XStream.html]

XStream


---


|  |  |  |
| --- | --- | --- |
| **Overview** \| **Package** \| **Class** \| **Tree** \| **Deprecated** \| **Index** \| **Help** \| **Weka's home** | |  |
| **PREV CLASS**   NEXT CLASS | **FRAMES**    **NO FRAMES**     **All Classes** |  |
| SUMMARY: NESTED \| FIELD \| CONSTR \| METHOD | DETAIL: FIELD \| CONSTR \| METHOD |  |


---


## weka.core.xml Class XStream

```
java.lang.Object
  weka.core.xml.XStream
```

**All Implemented Interfaces:**: RevisionHandler

---

``` public class XStream extends java.lang.Object implements RevisionHandler ```

This class is a helper class for XML serialization using
XStream .
XStream does not need to be present, since the class-calls are done generically via Reflection.

**Version:**
:   $Revision: 5562 $

**Author:**
:   Mark Hall (mhall{[at]}pentaho{[dot]}org)

---

| **Field Summary** | |
| --- | --- |
| `static java.lang.String` | `FILE_EXTENSION`             the extension for XStream files (including '.') |


**Constructor Summary**

`XStream()`


| **Method Summary** | |
| --- | --- |
| `static java.lang.Object` | `deSerialize(java.lang.String xmlString)`             Deserializes an object from the supplied XML string |
| `java.lang.String` | `getRevision()`             Returns the revision string. |
| `static boolean` | `isPresent()`             returns whether XStream is present or not, i.e. |
| `static java.lang.Object` | `read(java.io.File file)`             reads the XML-serialized object from the given file |
| `static java.lang.Object` | `read(java.io.InputStream stream)`             reads the XML-serialized object from the given input stream |
| `static java.lang.Object` | `read(java.io.Reader r)`             reads the XML-serialized object from the given Reader |
| `static java.lang.Object` | `read(java.lang.String filename)`             reads the XML-serialized object from the given file |
| `static java.lang.String` | `serialize(java.lang.Object toSerialize)`             Serializes the supplied object xml |
| `static boolean` | `write(java.io.File file, java.lang.Object o)`             write the XML-serialized object to the given file |
| `static boolean` | `write(java.io.OutputStream stream, java.lang.Object o)`             writes the XML-serialized object to the given output stream |
| `static boolean` | `write(java.lang.String filename, java.lang.Object o)`             writes the XML-serialized object to the given file |
| `static boolean` | `write(java.io.Writer writer, java.lang.Object toSerialize)`             writes the XML-serialized object to the given Writer |

**Methods inherited from class java.lang.Object**

`equals, getClass, hashCode, notify, notifyAll, toString, wait, wait, wait`

**Field Detail**

### FILE\_EXTENSION

```
public static final java.lang.String FILE_EXTENSION
```

:   the extension for XStream files (including '.')

    **See Also:**: Constant Field Values


**Constructor Detail**

### XStream

```
public XStream()
```


**Method Detail**

### isPresent

```
public static boolean isPresent()
```

:   returns whether XStream is present or not, i.e. whether the classes are in the
    classpath or not

    :   **Returns:**: whether XStream is available

---


### serialize

```
public static java.lang.String serialize(java.lang.Object toSerialize)
                                  throws java.lang.Exception
```

:   Serializes the supplied object xml

    :   **Parameters:**: `toSerialize` - the object to serialize **Returns:**: the serialized object as an XML string **Throws:**: `java.lang.Exception` - if something goes wrong

---


### write

```
public static boolean write(java.lang.String filename,
                            java.lang.Object o)
                     throws java.lang.Exception
```

:   writes the XML-serialized object to the given file

    :   **Parameters:**: `filename` - the file to serialize the object to: `o` - the object to write to the file **Returns:**: whether writing was successful or not **Throws:**: `java.lang.Exception` - if something goes wrong while writing to the file

---


### write

```
public static boolean write(java.io.File file,
                            java.lang.Object o)
                     throws java.lang.Exception
```

:   write the XML-serialized object to the given file

    :   **Parameters:**: `file` - the file to serialize the object to: `o` - the object to write to the file **Returns:**: whether writing was successful or not **Throws:**: `java.lang.Exception` - if something goes wrong while writing to the file

---


### write

```
public static boolean write(java.io.OutputStream stream,
                            java.lang.Object o)
                     throws java.lang.Exception
```

:   writes the XML-serialized object to the given output stream

    :   **Parameters:**: `stream` - the output stream: `o` - the object to write **Returns:**: true if everything goes ok **Throws:**: `java.lang.Exception`

---


### write

```
public static boolean write(java.io.Writer writer,
                            java.lang.Object toSerialize)
                     throws java.lang.Exception
```

:   writes the XML-serialized object to the given Writer

    :   **Parameters:**: `writer` - the Writer: `o` - the object to write **Returns:**: true if everything goes ok **Throws:**: `java.lang.Exception`

---


### read

```
public static java.lang.Object read(java.lang.String filename)
                             throws java.lang.Exception
```

:   reads the XML-serialized object from the given file

    :   **Parameters:**: `filename` - the file to deserialize the object from **Returns:**: the deserialized object **Throws:**: `java.lang.Exception` - if something goes wrong while reading from the file

---


### read

```
public static java.lang.Object read(java.io.File file)
                             throws java.lang.Exception
```

:   reads the XML-serialized object from the given file

    :   **Parameters:**: `file` - the file to deserialize the object from **Returns:**: the deserialized object **Throws:**: `java.lang.Exception` - if something goes wrong while reading from the file

---


### read

```
public static java.lang.Object read(java.io.InputStream stream)
                             throws java.lang.Exception
```

:   reads the XML-serialized object from the given input stream

    :   **Parameters:**: `stream` - the input stream **Returns:**: the deserialized object **Throws:**: `java.lang.Exception` - if something goes wrong while reading from stream

---


### read

```
public static java.lang.Object read(java.io.Reader r)
                             throws java.lang.Exception
```

:   reads the XML-serialized object from the given Reader

    :   **Parameters:**: `r` - the reader **Returns:**: the deserialized object **Throws:**: `java.lang.Exception` - if something goes wrong while reading from stream

---


### deSerialize

```
public static java.lang.Object deSerialize(java.lang.String xmlString)
                                    throws java.lang.Exception
```

:   Deserializes an object from the supplied XML string

    :   **Parameters:**: `xmlString` - the XML to deserialize from **Returns:**: the deserialized object **Throws:**: `java.lang.Exception` - if something goes wrong

---


### getRevision

```
public java.lang.String getRevision()
```

:   Returns the revision string.

    :   **Specified by:**: `getRevision` in interface `RevisionHandler`
    :   **Returns:**: the revision


---


|  |  |  |
| --- | --- | --- |
| **Overview** \| **Package** \| **Class** \| **Tree** \| **Deprecated** \| **Index** \| **Help** \| **Weka's home** | |  |
| **PREV CLASS**   NEXT CLASS | **FRAMES**    **NO FRAMES**     **All Classes** |  |
| SUMMARY: NESTED \| FIELD \| CONSTR \| METHOD | DETAIL: FIELD \| CONSTR \| METHOD |  |


---
